# An improved method of searching inferior parathyroid gland for the patients with papillary thyroid carcinoma based on a retrospective study

**DOI:** 10.3389/fsurg.2022.955855

**Published:** 2023-01-06

**Authors:** Shouyi Yan, Lanqin Lin, Wenxin Zhao, Bo Wang, Liyong Zhang, Shaojun Cai

**Affiliations:** ^1^ Fujian Medical University, Fuzhou, China; ^2^ Department of Thyroid Surgery, Fujian Medical University Union Hospital, Fuzhou, China; ^3^ The Department of General Surgery, Fujian Medical University Union Hospital, Fuzhou, China; ^4^ Minimal Invasive Center, Fujian Medical University Union Hospital, Fuzhou, China; ^5^ The Department of Anesthesia and Surgery, Fujian Medical University Union Hospital, Fuzhou, China

**Keywords:** parathyroid grand, inferior parathyroid gland, superior parathyroid gland, papillary thyroid cancer, thymus-related inferior parathyroid gland, thyroid surgery, parathyroid gland protection

## Abstract

**Objective:**

Many surgeons knew the importance of parathyroid gland (PG) in the thyroid surgery, but it was even more difficult to be protected. This study aimed at evaluating the effectiveness of the improved method of searching inferior parathyroid gland (IPG).

**Methods:**

213 patients were enrolled and divided into test and control groups according to different methods of searching IPG in the surgery. Consequently, we compared the surgical outcome parameters between the two groups, including the operative time, numbers of PG identifying (PG protection *in situ*, PG auto-transplantation, and PG accidental removal), numbers of the total lymph node (LN) and metastatic LN, parathyroid hormone (PTH), transient hypoparathyroidism, transient recurrent laryngeal nerve palsy, and postoperative bleeding.

**Results:**

We identified 194 (194/196, 98.98%) and 215 (215/230, 93.48%) PGs in the test group and control group, respectively, and there was a significant difference (*P* = 0.005), and this result was due to IPG identification differences (96/98, 97.96% vs. 100/115, 86.96%, *P* = 0.004). Meanwhile, there was a lower ratio of IPG auto-transplantation in the test group compared with that in the control group (46.94% vs. 64.35%, *P* = 0.013). Serum PTH one day after the operation was 3.65 ± 1.86 vs. 2.96 ± 1.64 (*P* = 0.043) but with no difference at 6 months. There were no differences in metastatic LN and recurrent laryngeal nerve palsy between two groups.

**Conclusion:**

The improved method of searching IPG was simple, efficient, and safe, which was easy to be implemented for searching IPG and protecting it well.

## Introduction

Thyroid carcinoma (TC) was the most common endocrine malignancy, and its incidence rates had been increasing over the past 30 years (1). In 2012, 298,000 new thyroid cancer cases and 40,000 thyroid cancer deaths were found all over the world. The percentage of new TC cases and TC deaths in China accounted for 15.6% and 13.8% of the world, respectively (2). The predominant treatment for TC was surgery, and the key point of thyroid surgery was to protect the recurrent laryngeal nerve (RLN) and parathyroid gland (PG). Now finding RLN had become easier with the help of intraoperative neuromonitoring (IONM) (3), while there were still many difficulties in the functional protection of PG.

The incidence of permanent hypoparathyroidism was around 1.5%, which would seriously affect the quality of patient's living standards, including numbness of patient's hands and feet, osteoporosis, and other problems (4, 5). The difficulty of PG protection was to identify PG promptly, especially for the inferior parathyroid gland (IPG) during surgery. As far as we knew, the IPG were closely related to thymus in the process of human embryonic development (6); thus, we presumed that thymus is a key factor for searching the IPG retrogradely. Secondly, we assumed that an improved method would help search the IPG effectively and protect it well.

## Material and methods

### Patients’ characteristics

A total of 213 patients were enrolled in this study from August 2019 to December 2020, and their characteristics are shown in Table 1. All the patients were diagnosed with unilateral papillary thyroid carcinoma (PTC) by preoperative fine needle aspiration. According to different methods of searching IPG, the patients were separated into the test group (*n* = 98) and the control group (*n* = 113). According to the application time of the improved way of searching IPG, which was used on the patients at the point of August 1, 2019. Therefore, the test group was defined with the use of the improved way of searching IPG, and control group was defined without it. The inclusion criteria were as follows: (1) The longest PTC diameter was less than 4 cm; (2) The patients were confirmed as unilateral PTC. Additionally, the exclusion criteria were as follows: (1) Tumor had invaded IPG and surrounded tissue obviously; (2) The patients had a history of thyroid surgery; (3) The patients were less than 16 years old; (4) The patients were unable to comply with the follow-up; (5) There were metastatic lymph nodes in the preoperative examination. All the operations were performed by the same surgeon, and all the patients underwent thyroid lobectomy plus dissection of the central lymph node. The study was approved by the Ethics Committee of FuJian Medical University Union Hospital.

**Table 1:** Patients’ characteristics.

Characteristics	Test group (98 cases)	Control group (115 cases)	*P*-value
Age	42.4 ± 11.85	40.5 ± 11.25	0.829
Sex (male /female)	74/24	85/30	0.913
Size of tumor	0.80 ± 0.32	0.82 ± 0.40	0.534
Tumor microinvasion	24/98	29/115	1.000
Ratio of IONM	15/98	20/115	0.823

IONM, intraoperative neuromonitoring.

### Surgical procedure of thyroid lobectomy plus dissection of the central lymph node

#### Improved method of searching IPG in the test group

**Figure 1:**
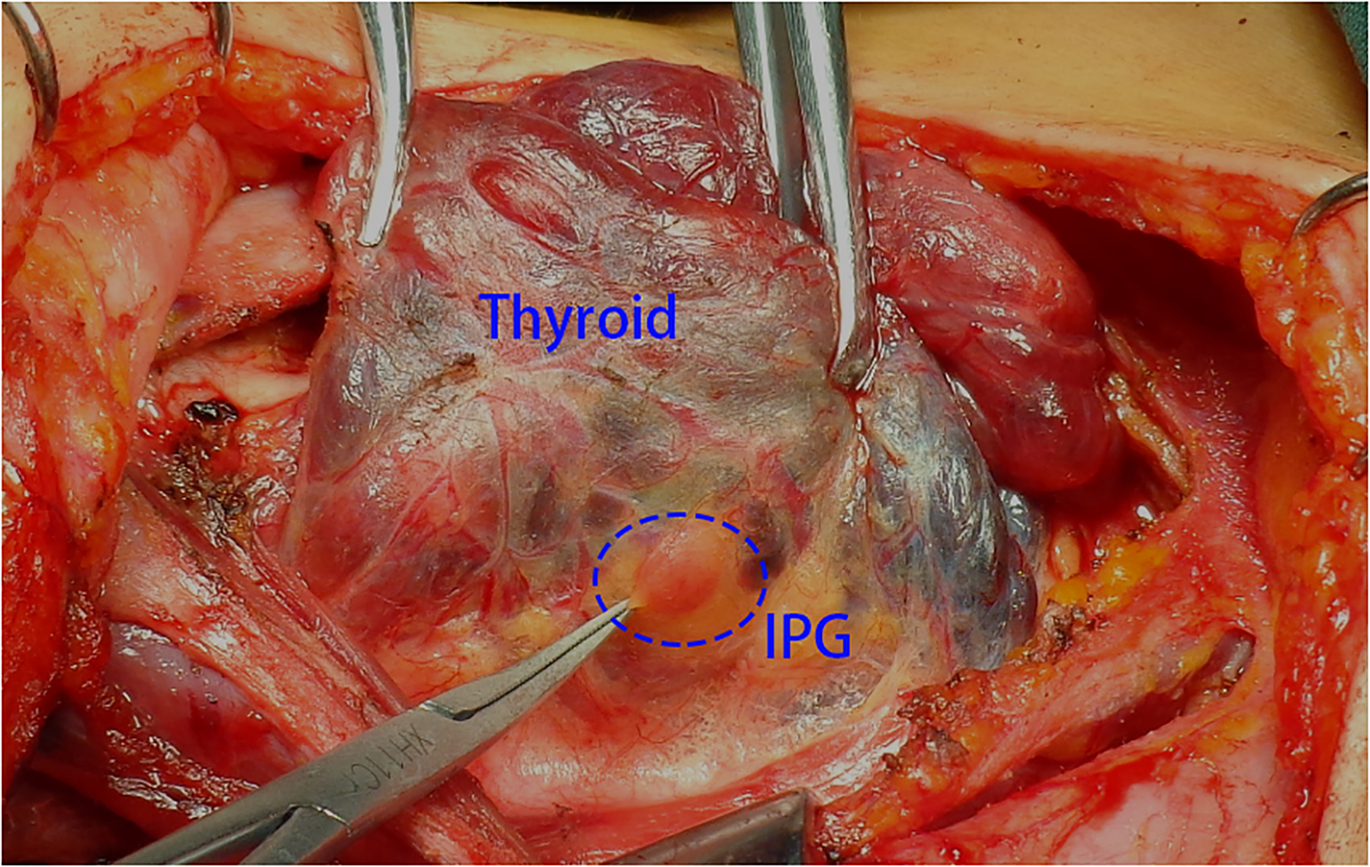
The thyroid gland was pulled to the contralateral side and the IPG was observed in the lower part of the thyroid gland. IPG, inferior parathyroid gland.

**Figure 2:**
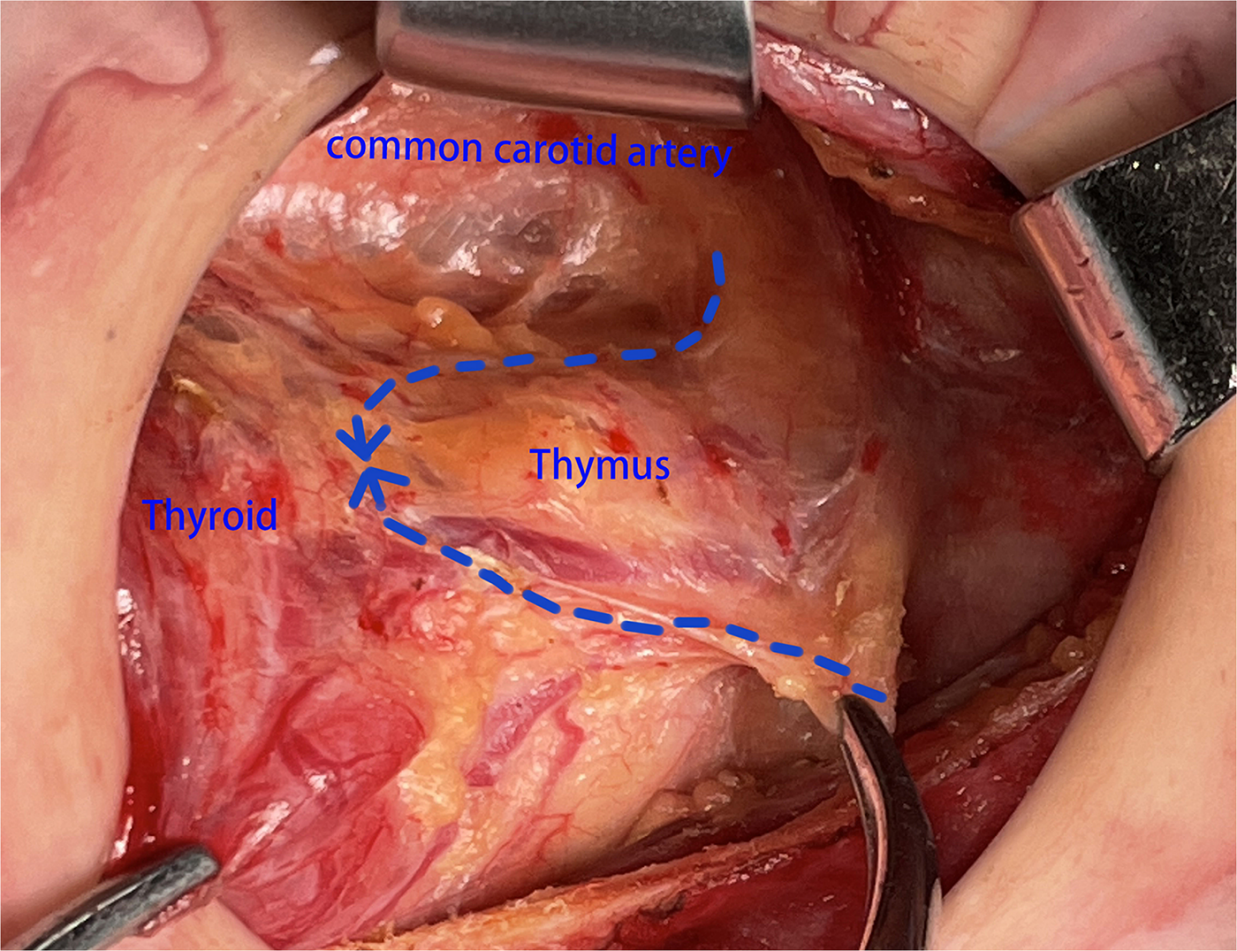
The thymus was found and separated from the tail to head side.

**Figure 3:**
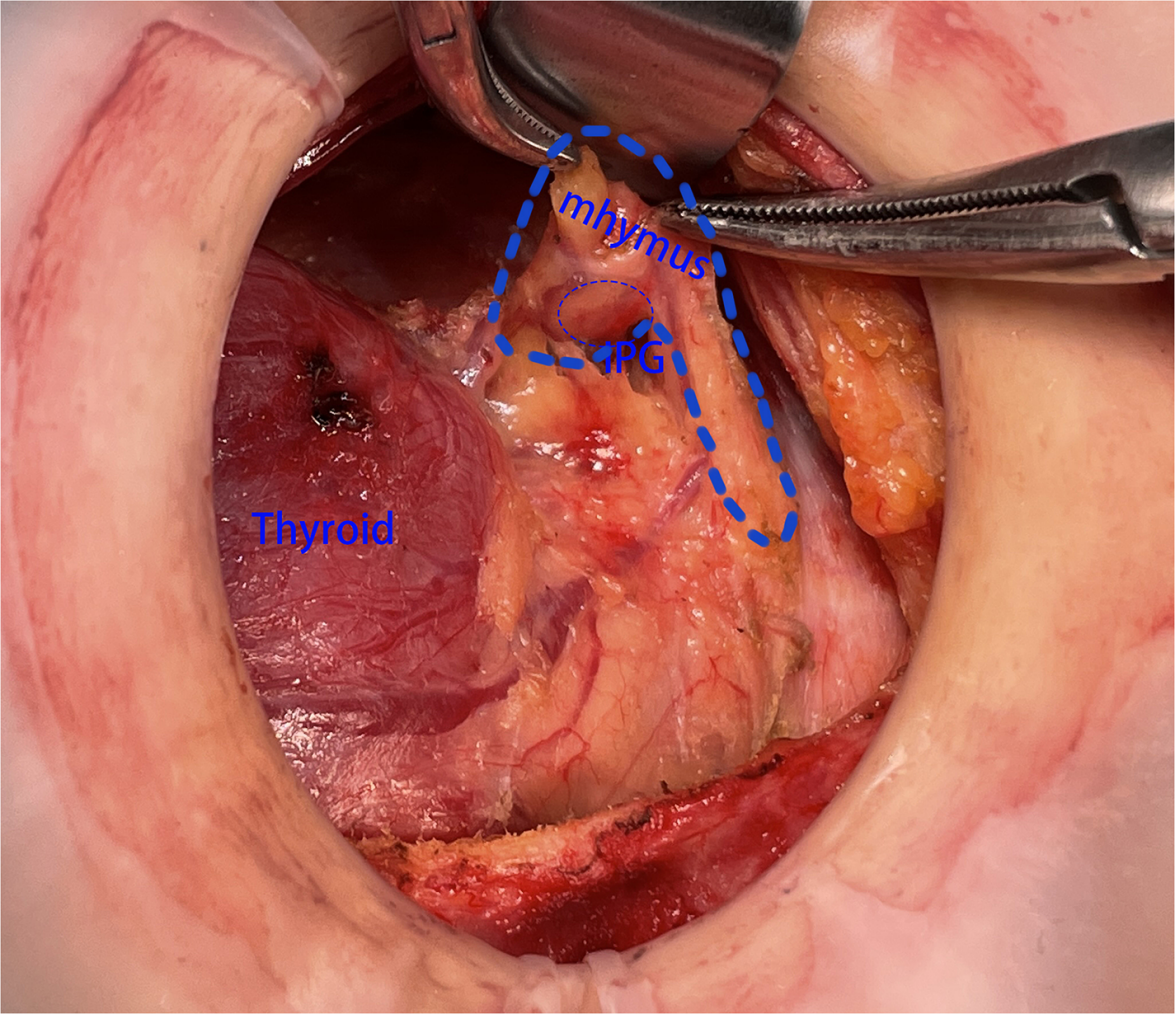
The SPG was found to be located within the top of the thymus. SPG, superior parathyroid gland.

Step 1: To protect the superior parathyroid gland (SPG), we severed the thyroid isthmus and subsequently the anterior branch of the superior thyroid artery along the cricothyroid gap, which was near to where the RLN entered the larynx. The lateral side of the thyroid gland was then dissected, exposing the common carotid sheath up to the top pole of the thyroid and down to the thymus.
Step 2: We pulled the thyroid gland to the contralateral side and observed the IPG in the lower part of the thyroid gland. If the IPG was attached closely to the thyroid and was difficult to be retained *in situ*, prompt transplantation was needed (Figure 1).
Step 3: According to the previous report, we rapidly exposed the RLN during surgery and located the thymus in the lower portion of the common carotid artery, where the thymus’ nourishing blood arteries must be preserved. Following the protocol, we first dissociated the thymus along its capsule to expose the majority of the thymus on the tumor sides of the trachea. Next, we removed the lymphoid tissue covering the superficial surface of the thymus and freed the deep surface of the thymus to assist the subsequent lymph node dissection. After the aforementioned procedure, the thymus in the neck could be clearly seen. We then continued to dissociate the thymus from the distal caudal lobe of the thymus (Figure 2) and did not attempt to sever the caudal lobe of the thymus during the process of separation, as there were numerous caudal lobes of thymus in the neck, and the inferior thyroid may be in or closely associated with one of the caudal lobes of thymus. Part of the inferior parathyroid gland may be situated in the thymus or distal to the caudal lobe of the thymus. During this procedure, it is possible that a portion of the IPG was located in the thymus or distal to the caudal lobe of the thymus (Figure 3).

**Figure 4:**
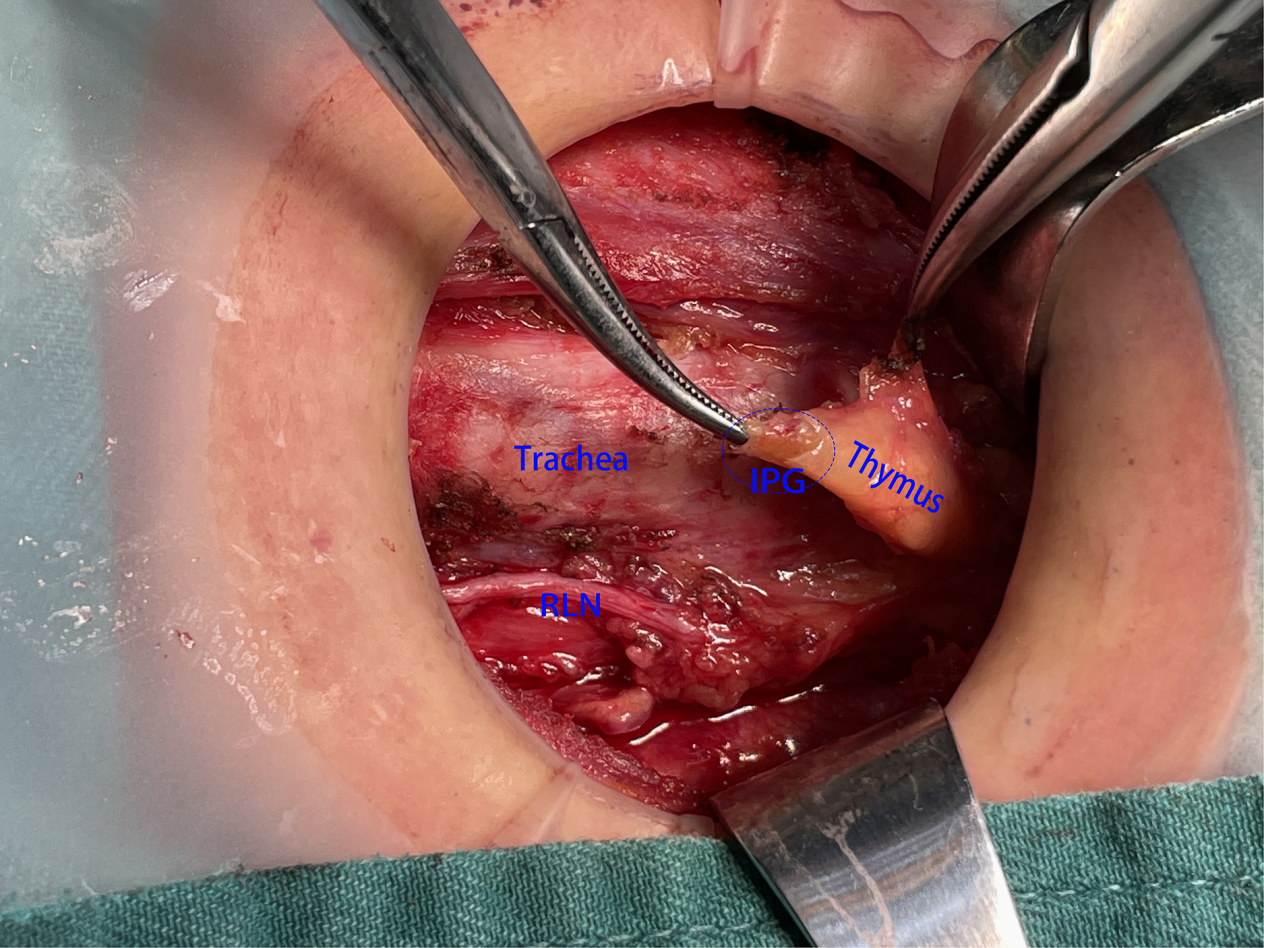
SPG was wrapped by the thymus, and it was difficult to be identified directly and clearly with the naked eye unless the capsule of the thymus was opened. SPG, superior parathyroid gland.

**Figure 5:**
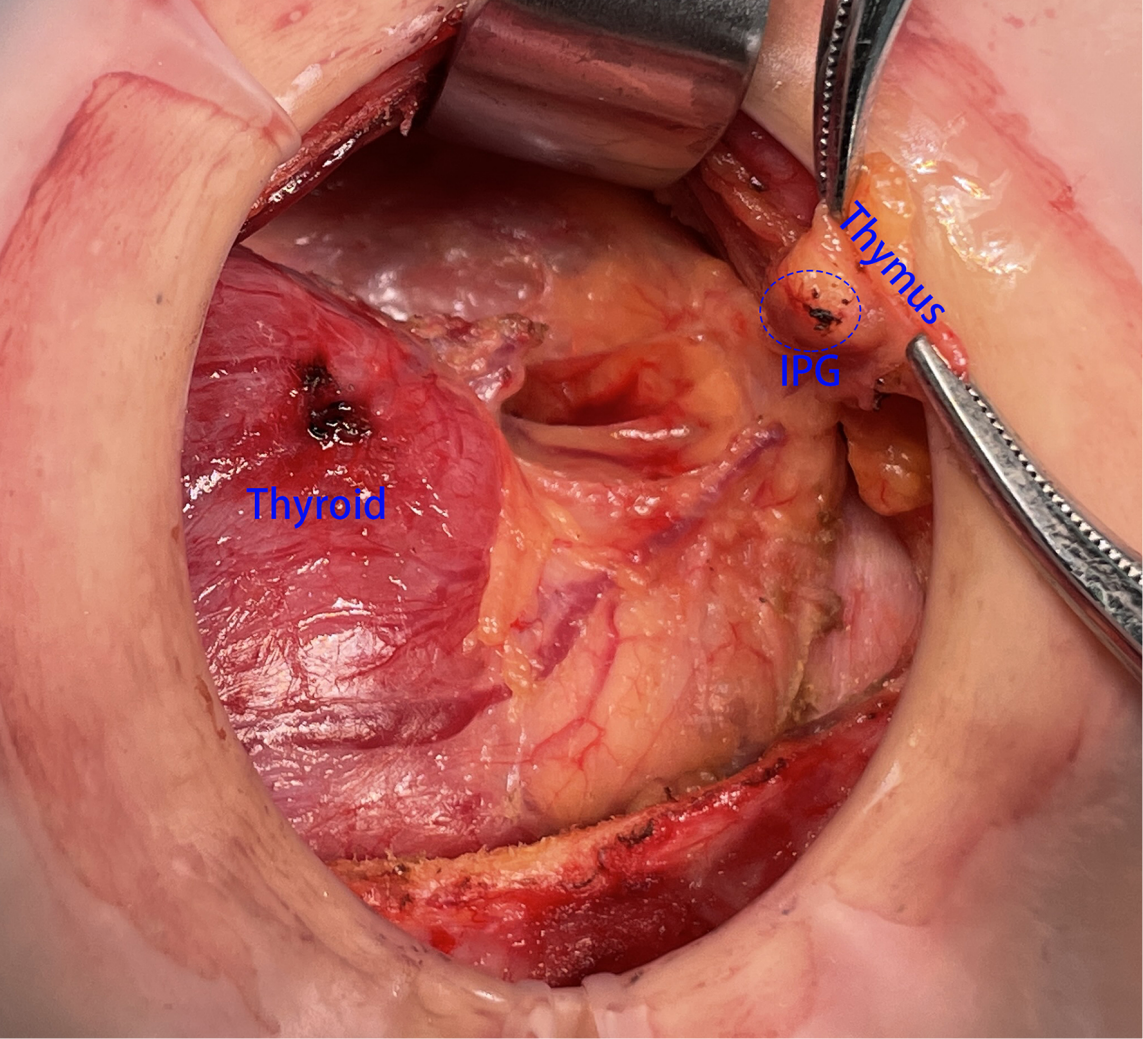
SPG and thymus could be *in situ* reservation as an organic whole. SPG, superior parathyroid gland.

However, there were also some patients in whom IPG was wrapped by thymus, and it was difficult to be identified directly and clearly by naked eye (Figure 4). If IPG was not observed, we should continue separating the thyroid thymus ligament along the direction of the thyroid. Generally, IPG would be found within 10 mm distance from the top of the thymus. At this time, IPG and thymus could be *in situ* reserved as a whole (Figure 5). Nevertheless, if we found IPG far away from the top of the thymus (greater than 10 mm) or IPG and thymus were found free significantly, prompt transplantation of IPG was required.

Step 4: We continued with lobe thyroidectomy and central lymph node dissection. If the IPG was not detected with the above-mentioned process, looking for IPG in the excised material was required.

#### Searching SPG in the control group

Step 1, step 2, and step 4 were same in both groups, and we focused on step 3 with a detailed statement. We dissected the lower pole of the thyroid, separated, and exposed the RLN to the position where RLN entered the larynx. Then, we severed the lower pole of the thyroid and completed thyroidectomy and central lymph node dissection. If the IPG was found during the operation, it would be retained *in situ* together with the surrounded non-lymph node tissue. If the IPG could not be retained *in situ*, it would be implanted in the left forearm promptly.

### Identification and protection of PG during the operation

It was known that the anatomic location of the SPG was fixed at the dorsal side of upper pole of the thyroid lobe at the level of the inferior border of the cricoid cartilage, but the location of IPG was variable due to its embryologic relationship to the thymus (8). Ordinarily, the key point of PG identification was to observe the blood supply (microvessel on the surface of PG), texture (the hardness range is between fat and lymph nodes), color (light brown), and neighboring relationship (usually connected with the thymus) (9). To preserve the PG at its original location was necessary when we discovered it during the process. If the PG lacked blood flow or was thymus-free, it should be put into the forearm in a timely manner. Also, if the PG was not found during the operation, we should look for it in the removed specimens according to its physical characteristics, which could be confirmed by parathyroid hormone (PTH) test and transplanted into the forearm promptly.

### PTH rapid test of the PG in all the patients we found

**Figure 6:**
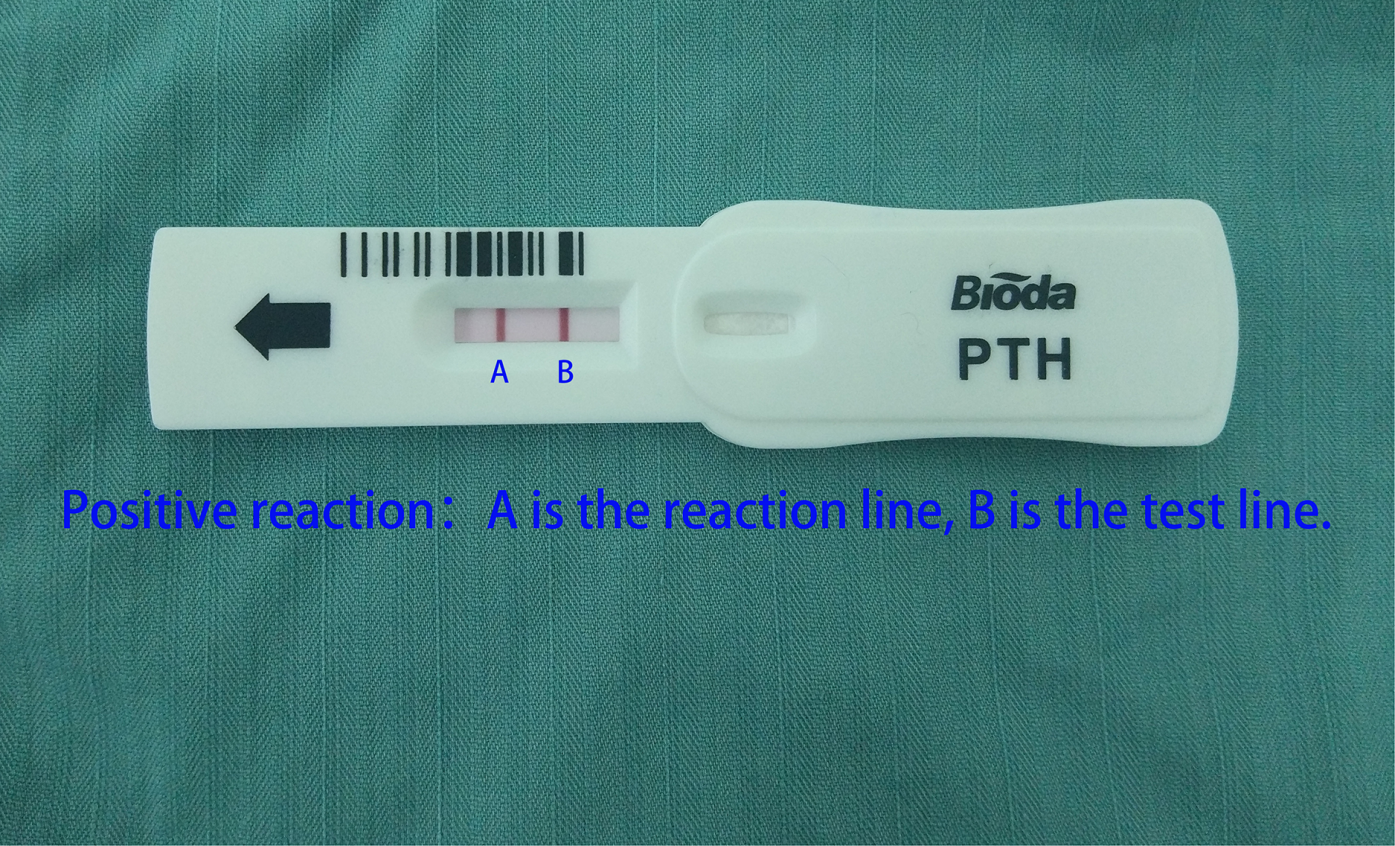
The suspected parathyroid tissue was punctured 3–5 times with a 1 ml needle.

**Figure 7:**
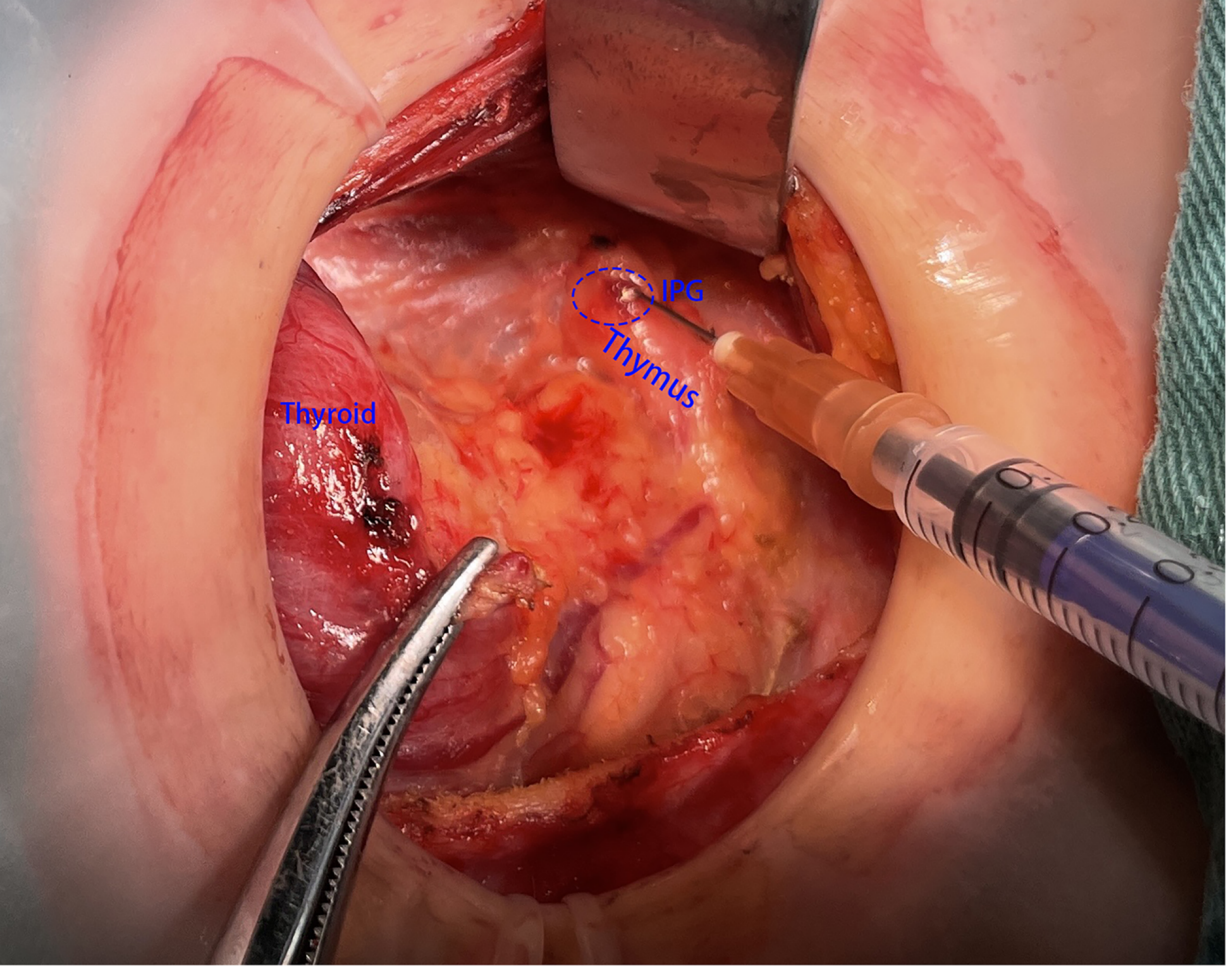
Display of the reaction line when the parathyroid tissue was confirmed.

After properly rinsing the needle in 1 ml saline, it was used to pierce the suspected parathyroid tissue three to five times (Figure 6). After all else was done, 100 µl of liquid was poured into the detection hole of the PTH quick test paper. Figure 7 shows a colored reaction line if the parathyroid tissue was positive, and a colorless line if it was negative.

### Follow-up and postoperative treatment of hypoparathyroidism

The parathyroid hormones level would be tested in 1 day and 6 months after the operation. Postoperative hypoparathyroidism was defined when the PTH was less than 1.3 mmol/L after 6 months. The follow-up time was 12–20 months for the patients. Calcium supplementation was not routinely administered to the patients, but calcium and vitamin D were routinely prescribed with symptomatic hypoparathyroidism until the level of PTH recovered. Intravenous substitution of calcium was not routine unless serious symptomatic hypocalcemia appeared. Levothyroxine treatment was necessary for all patients after the operation, Additionally, TSH levels within the normal range (0.5–2.0 mIU/L) were considered to be within acceptable parameters.

### Data collection

General characteristics, intraoperative factors, pathologic examination, the number of LNs and metastatic LNs in the resected specimens, and postoperative complications were collected retrospectively. Accidental removal of PG was defined without finding the PG during operation (including careful inspection of the resected specimens) but finding it in the postoperative paraffin pathology. The primary checkpoints were the operative time, number of PG identified, PG protection *in situ*, PG auto-transplantation and PG accidental removal, number of the total LNs and metastatic LNs, PTH level, transient hypoparathyroidism, transient recurrent laryngeal nerve palsy, and postoperative bleeding.

### Statistical analysis

Statistical analysis was performed by SPSS 26.0, Chicago, IL, United States. All values were presented as mean ± standard deviation. A *t*-test or Chi-square test was used to determine statistical significance, requiring *P* < 0.05 was statistically significant.

## Results

### Patients characteristics

The clinical characteristics of the patients in two groups were summarized in Table 1. PTC was confirmed by the postoperative pathology for all the patients. There were no significant differences between the two groups in terms of age (*P* = 0.829), sex (*P* = 0.913), tumor sizes (*P* = 0.534), extrathyroidal extension (*P* = 1.000), and ratio of IONM (*P* = 0.823) (Table 1).

### The identifying and protecting of PG (thymus-related PG)

Test group (2/98) and control group (15/115) lacked IPG. Postoperative paraffin pathology results also revealed that 15 PG of accidental removal occurred in the control group, whereas 2 PG in the test group. The difference was statistically significant (*P* = 0.004) (Table 2).

**Table 2:** The identification and protection of PG (thymus-related PG).

Variable	Test group (98 cases)	Control group (115 cases)	*P*-value
Operative time* (min)	25.02 ± 3.15	30.53 ± 3.55	0.000
Numbers of LN (CND)	11.50 ± 2.65	11.25 ± 2.60	0.836
Metastatic LN (CND)	4.50 ± 1.45	4.55 ± 1.55	0.762
Identifying PG	194/196	215/230	0.005
Identifying SPG	98/98	115/115	/
Identifying IPG	96/98	100/115	0.004
PG protection *in situ*	120/196 (61.22)	109/230 (47.39)	0.005
SPG protection *in situ*	70/98 (71.43)	83/115 (72.17)	1.000
IPG protection *in situ*	50/98 (51.02)	27/115 (23.47)	0.000
PG auto-transplantation	74/196 (37.76)	106/230 (46.09)	0.094
SPG auto-transplantation	28/98 (28.57)	32/115 (27.83)	1.000
IPG auto-transplantation	46/98 (46.94)	74/115 (64.35)	0.013
PG of accidental removal	2/98	15/115	0.004
Transient hypoparathyroidism	3/98	4/115	1.000
postoperative bleeding	0/98	1/115	1.000
PTH level (preoperation)	5.65 ± 2.20	5.74 ± 2.32	0.874
PTH level (pmol/L, 1 day)	3.65 ± 1.86	2.96 ± 1.64	0.003
PTH level (pmol/L, 6 months)	5.30 ± 1.52	5.16 ± 1.42	0.062
Serum calcium (pmol/L, 1 day)	2.45 ± 0.20	2.42 ± 0.19	0.942
Serum calcium (pmol/L, 6 months)	2.38 ± 0.17	2.36 ± 0.17	0.866
Transient recurrent laryngeal nerve palsy.	2/98	3/115	1.000
Transient recurrent laryngeal nerve palsy	0/98	1/115	1.000

PG, parathyroid gland; LN, lymph node; SPG, superior parathyroid gland; IPG, inferior parathyroid gland; PTH, parathyroid hormone.

The normal range for PTH values is 1.3–9.3 pmol/L, The normal range for calcium is 2.1–2.7 mmol/L.

We identified 194 (194/196, 98.98%) and 215 (215/230, 93.48%) PG in the test group and control group, respectively, and there was a significant difference (*P* = 0.005); because all the SPG were identified in the two groups, this difference was only found in the IPG identification (96/98, 97.96% vs. 100/115, 86.96%) with a significant difference (*P* = 0.004). In the meantime, the ratio of IPG auto-transplantation was lower in the test group than in the control group (46.94% vs. 64.35%) (*P* = 0.013). No significant difference was discovered between the two groups in terms of SPG auto-transplantation (28/98, 28.57% vs. 32/115, 27.83%, *P* = 1.000). In addition, it was discovered that the PTH level (1 day) in the test group was significantly greater than that of the control group (3.651.86 vs. 2.961.64) (*P* = 0.003). However, there was no difference between the right hand PTH level and serum calcium level (6 month).

### Serum calcium and parathyroid levels in the patients with PG transplantation in the arm

There was a higher successful rate of IPG auto-transplantation in the test group than that in the control group (87.84% vs. 74.53%), with a major difference (*P* = 0.001). It was also found that the PTH level in the left hand (6 months) in the test group was higher than that in the control group (35.46 ± 20.65 vs. 23.46 ± 12.54) with an obvious difference (*P* = 0.001). However, there was no statistical difference in the aspect of PTH level in the right hand (2 and 6 months) and left hand (2 month) (Table 3).

**Table 3:** Serum calcium and parathyroid levels in the patients with PG transplantation in the arm.

Variable	Time points of postoperative review	Test group (74 patients)	Control group (106 patients)	*P*-value
SPG auto-transplantation	—	28/74	32/106	—
IPG auto-transplantation	—	46/74	74/106	—
PTH level in the left hand	2 weeks	7.88 ± 2.75	6.98 ± 2.92	0.675
	6 months	35.46 ± 20.65	23.46 ± 12.54	0.001
PTH level in the right hand	2 weeks	5.25 ± 1.48	5.17 ± 1.39	0.524
	6 months	5.46 ± 1.55	5.39 ± 1.46	0.673
Success rate of PG auto-transplantation	6 months	65/74 (87.84%)	79/106 (74.53%)	0.001

PG, parathyroid gland; SPG, superior parathyroid gland; IPG, inferior parathyroid gland; PTH, parathyroid hormone.

Success rate of PG transplantation was defined as the parathyroid hormone in the left hand/that in the right hand ≥2 in the postoperative 6 months (10).

### Central lymph node dissection in the two groups

All the patients underwent dissection of the central lymph node, but there was no statistically significant difference between the two groups in the aspect of total LNs and metastasis LNs (*P* = 0.836, *P* = 0.762). However, there was less operative time in the test group compared with that in the control group (25.02 ± 3.15 vs. 30.53 ± 3.55) with a major difference (*P* = 0.000) (Table 2).

### Side effects and operative complications

In this study, some patients encountered the complications including postoperative bleeding (1/213), transient hypoparathyroidism (7/213), transient recurrent laryngeal nerve palsy (5/213), and permanent recurrent laryngeal nerve palsy (1/213) with no statistical difference in two groups (*P* < 0.05). The patients with transient hypoparathyroidism did not show low-calcium symptoms and the PTH level recovered after 2 weeks. Also, there was only one patient with transient vocal cord dyskinesia recovered after 6 months according to the electronic laryngoscope (Table 2).

## Discussion

Lobe thyroidectomy plus central lymph node dissection (CND) was a preferred treatment for the patients with PTC in China (11, 12). An increasing number of surgeons had paid much attention in the protection of PG and RLN to prevent permanent hypoparathyroidism and nerve palsy, which were the most common complications in the thyroid surgery (13, 14). Now finding RLN was no longer a big problem, particularly for the experienced surgeons and with the help of IONM (15). However, there was still plenty of room for improvement in PG protection. As we know that the SPG was relatively fixed anatomically, and it was easy to be found and retained *in situ* in the surgery. So, the difficulty was the IPG functional protection, especially its *in situ* preservation. The Chinese recommendations described the link between the PG and the thyroid gland (16), but this information was not useful in IPG searching during surgery, particularly when the IPG was encased by the thymus. The old method of searching for IPG depended mostly on the physicochemical properties of the parathyroid gland, but it was affected by a number of variables, including surgical expertise, obesity, PG fatty degeneration, and intraoperative bleeding (17, 18). In conclusion, a straightforward and effective method of searching IPG was necessary.

As we knew that IPG was pulled down by the thymus during embryonic development (19), so the IPG might appear in any pathway of thymus migration process. In other words, most IPG was related to the thymus anatomically, in which case we called it thymus-related inferior parathyroid gland (TRIPG) (7). In our procedure, the thymus was easily located on the superficial surface of the common carotid artery in the lower neck. In addition, we could dissect it from the caudal to the cephalic end. In the method described above, the IPG may be located at the top of the thymus. However, in some patients, the IPG was wrapped by thymus tissue and not visible to the naked eye directly and obviously. Meanwhile, if the IPG was not found at the top of the thymus, we should continue to separate it along the continuous thyroid thymus ligament toward the thyroid, and the IPG could be found within 10 mm away from the top of thymus. Then, IPG and thymus were lumped together for preservation *in situ*. Studies revealed that PG auto-transplantation may sustain its function; therefore, if the IPG was far from the thymus's apex, we chose a more aggressive transplant. We believe IPG with thymus tissue blood supply will survive.

Nonetheless, there was a disadvantage in our study that no methods were available for verifying the blood supply of the IPG. We speculated its blood supply was mainly from the microcirculation of the adjacent thymus, but there was no research data to prove, which may be addressed by the intraoperative validation with indolephilophilic green (20).

In this study, IPG recognition in the test group (96/98, 97.96%) were higher in the test group than that of the control group (100/115, 86.96%) with a significant difference (*P* = 0.004). Meanwhile, there was a lower ratio of IPG auto-transplantation in the test group compared with that in the control group (46.94% vs. 64.35%, *P* = 0.013). It showed this improved way was more efficient in the IPG recognition and retention *in situ*. Whereas in the control group, the IPG was distinguished with difficulty from the fat and lymph nodes in the central lymphatic adipose tissue due to the physical similarity between the PG and surrounding tissue especially in those who were obese. At the same time, the PTH level was higher than that of the control group on the first day after operation, which means that this improved method may protect the IPG well *in situ*. It was reported that the activity of parathyroid tissue decreased significantly after more than 30 min *in vitro* (10), and we also found that there was a higher successful rate of IPG auto-transplantation in the test group than that in the control group (87.84% vs. 74.53%), with a major difference (*P* = 0.001). Meanwhile, the PTH level in the left hand and successful ratio of PG transplantation in the test group was higher than that in the control group in the 6-month follow-up; it might be related to the IPG timely intraoperative transplantation, which was more conducive to the survival of transplanted PG.

In the meantime, neither group contained a patient with permanent laryngeal recurrent nerve damage and hypoparathyroidism. Six months later, one patient with transient laryngeal recurrent nerve injury had recovered, and there was no difference between the two groups. Therefore, the enhanced method for locating IPG was safe and practical, which may be attributed to the following factors: (1) Gaps between thymus and its surrounding adipose tissue was loose and easy to be separated for lacking vascular tissue. (2) We used a new approach to expose RLN, which was in a visible and safe position before we searched the IPG. Meanwhile, previous surgical procedure could provide safety in the process of IPG separation by avoiding damage to the nearby RLN due to thermal damage, pull or clamps, etc., especially in the patients without protection of IONM. (3) The improved way would decrease the difficulty of IPG identification by reducing the bleeding and exudation, which could be verified by less operative time and postoperative drainage volume in the test group. Finally, before looking for the IPG, we had exposed the RLN completely, and central lymph node dissection was completed more efficiently and quickly.

Main purpose of this method was to look for IPG based on thymus, and it was simple and safe, especially suitable for the elderly patients with obesity and short and coarse neck. Because the elderly often merge parathyroid steatosis, and it was difficult to distinguish PG from adipose tissue in excised specimen.

Auto-transplantation of PGs has been shown to maintain their function (10), but realizing the quick PG search requires a fixation approach first. Based on the “layer of thymus-blood vessel-inferior parathyroid gland” idea, Wang et al. presented a method for determining whether or not the inferior parathyroid gland received adequate blood flow (21). Wang et al. also dissected the thyrothymic ligament meticulously in order to examine the inferior parathyroid gland (22). Since we think that IPG *in situ* with blood supply from thymus tissue are more likely to survive, our research focused on searching IPG through the thymus retrograde and deciding whether IPG should retain *in situ* based on the anatomical relationship between parathyroid and thymus. Therefore, this method could greatly reduce the difficulty of searching the IPG and increase the chance of survival of transplanted PG which cannot be retained *in situ*. However, this study also had the following disadvantages: (1) Only unilateral thyroidectomy was involved, so the serum PTH level after surgery may be affected by the contralateral PG. (2) The survival of thymus-associated parathyroid glands could not be verified after surgery, so further research should be carried out at a later stage. (3) It was not useful for the IPG recognition when IPG was in the thyroid gland, so intraoperative searching of excised specimen was still required in order to look for IPG, especially when there was no IPG found intraoperatively. (4) The number of cases was small, and a large number sample study with multiple centers was recommended. (5) This anatomical connection was used in the search and preservation of IPG during thyroid surgeries performed on or after August 1, 2019. However, grouping based on this date is indeed affected by several confounding factors, which will be further solved through the subsequent prospective study.

## Conclusion

Based on the clinic practice mentioned above, we believed the improved method of searching IPG was simple and feasible with lower risk of undermining RLN and PG, which could be useful for an inexperienced surgeon.

## Data Availability

The original contributions presented in the study are included in the article/Supplementary Material, further inquiries can be directed to the corresponding author.
